# Stem Cell Therapy for Autism Spectrum Disorder: A Systematic Review and Meta-Analysis of Clinical Evidence

**DOI:** 10.1192/j.eurpsy.2026.11127

**Published:** 2026-07-31

**Authors:** S. M. M. Fekrat, V. Sharma, S. Prasad, S. Gunturu

**Affiliations:** 1 Kabul University of Medical Sciences, Kabul, Afghanistan; 2 Dayanand Medical College & Hospital Ludhiana, Ludhiana, India; 3 Psychiatry, BronxCare Health System, New York, United States

## Abstract

**Introduction:**

Autism spectrum disorder (ASD) affects approximately 1 in 36 children globally, with limited evidence-based treatments addressing core symptomatology. The emerging regenerative medicine approaches using stem cell therapy have shown potential for modulating neuroinflammation and enhancing neuroplasticity in neurodevelopmental conditions. However, the clinical efficacy and safety profile of stem cell interventions for ASD remains uncertain.

**Objectives:**

This study aims to perform a systematic review and meta-analysis of clinical studies evaluating the efficacy and safety of stem cell therapy in children with autism spectrum disorder, as well as to identify optimal treatment protocols and patient selection criteria.

**Methods:**

A comprehensive systematic review was conducted in accordance with the PRISMA guidelines across multiple databases (PubMed, Scopus, PsycINFO) on September 17, 2025, yielding a total of 1,698 records (Figure 1). After removing 399 duplicates, the remaining 1299 records were screened based on title, abstract, and full-text analysis. Inclusion criteria: (1) randomized controlled trials, controlled clinical trials, and open-label studies; (2) participants diagnosed with ASD using standardized criteria (DSM-5, ADOS, ADI-R); (3) stem cell interventions (mesenchymal stem cells, umbilical cord blood, neural stem cells); (4) standardized outcome measures. Exclusion criteria: preclinical studies, case reports, and non-English publications. Two independent reviewers conducted data extraction and quality assessment (Risk of Bias). Cochrane RevMan 5.4 was used for meta-analysis.

**Results:**

All 22 studies demonstrated the efficacy of stem cell therapy in the treatment of ASD. Autism Spectrum Disorder was diagnosed according to the DSM-5 TR criteria. The Childhood Autism Rating Scale (CARS), Autism Treatment Evaluation Checklist (ATEC), Vineland Adaptive Behavior Scales (VABS), Aberrant Behavior Checklist (ABC), and Pervasive Developmental Disorder Behavior Inventory T-score (PDDBI) were used to evaluate autism at various durations during clinical trials. A statistically significant overall improvement in ASD was seen post-treatment with stem cells, on meta-analysis, with a pooled standard mean difference (SMD) of -1.03 [95% CI (-1.23, -0.83), Z=10.03, p<0.00001] but with high heterogeneity (I2= 95%) (Figure 2). Few studies also found stem cell therapy to be more effective than placebo, with SMD of -4.99 [95% CI (-8.11, -1.88), Z=3.14, p=0.002] with low heterogeneity (I2=0%) (Figure 3).

**Image 1:**
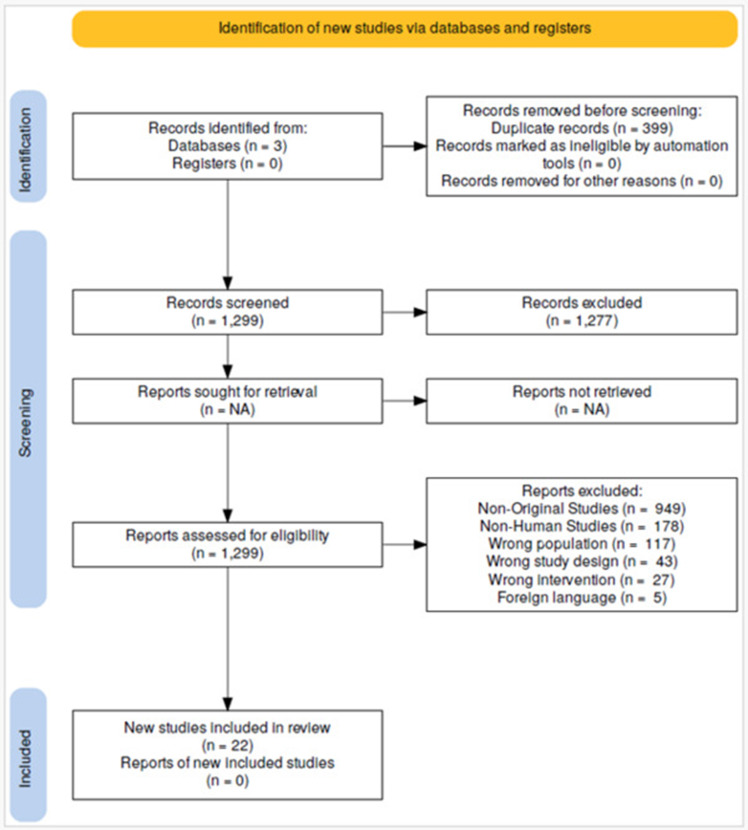
Image 1: Long description.

**Image 2:**
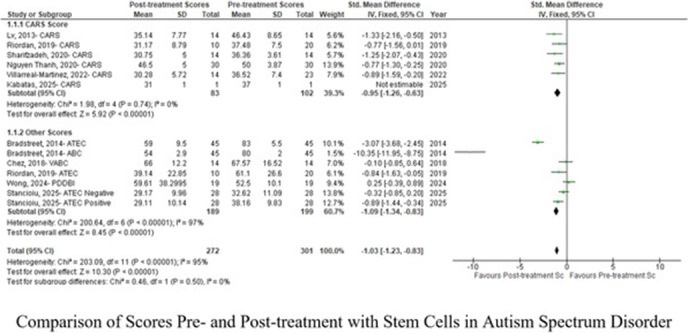
Image 2: Long description.

**Image 3:**
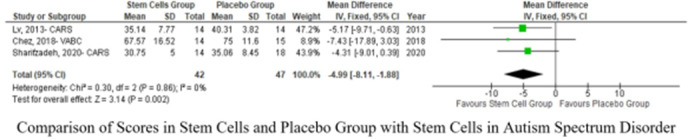
Image 3: Long description.

**Conclusions:**

The existing literature exhibits the efficacy of stem cell therapy as a treatment for autism spectrum disorder. Further clinical trials of stem cells in varied geographical locations and minorities with ASD should be spearheaded to assess the efficacy in all populations. Existing protocols for the treatment of ASD should be modified to reflect our findings.

**Disclosure of Interest:**

None Declared

## Long descriptions

### Image 1: Long description

A flowchart organized into three vertical phases: Identification, Screening, and Included.

1. Identification Phase:

- Top box: Identification of new studies via databases and registers.

- Left box: Records identified from Databases (n = 3) and Registers (n = 0).

- Right box (Exclusions): Records removed before screening: Duplicate records (n = 399), Records marked as ineligible by automation tools (n = 0), and Records removed for other reasons (n = 0).

2. Screening Phase:

- First central box: Records screened (n = 1,299). An arrow points right to Records excluded (n = 1,277).

- Second central box: Reports sought for retrieval (n = N A). An arrow points right to Reports not retrieved (n = N A).

- Third central box: Reports assessed for eligibility (n = 1,299). An arrow points right to a detailed exclusion box.

- Exclusion details: Reports excluded: Non-Original Studies (n = 949), Non-Human Studies (n = 178), Wrong population (n = 117), Wrong study design (n = 43), Wrong Intervention (n = 27), and Foreign language (n = 5).

3. Included Phase:

- Final bottom box: New studies included in review (n = 22) and Reports of new included studies (n = 0).

Navigate back to Image 1:.

### Image 2: Long description

The forest plot is divided into two sections.

Section 1.1.1 C A R S Score:

Includes six studies: Lv 2013, Riordan 2019, Sharifzadeh 2020, Nguyen Thanh 2020, Villarreal-Martinez 2022, and Kabatas 2025. The Std. Mean Difference for most studies falls between -2 and 0, favoring post-treatment. The subtotal for this group shows a diamond at -0.95 with a 95% C I of -1.26 to -0.63. Heterogeneity: Chi-squared = 1.98, I-squared = 0%.

Section 1.1.2 Other Scores:

Includes seven entries: Bradstreet 2014 A T E C, Bradstreet 2014 A B C, Chez 2018 V A B C, Riordan 2019 A T E C, Wong 2024 P D D B I, Stancioiu 2025 A T E C Negative, and Stancioiu 2025 A T E C Positive. Bradstreet 2014 A B C is a significant outlier with a Std. Mean Difference of -10.35. The subtotal diamond is at -1.09 with a 95% C I of -1.34 to -0.83. Heterogeneity: Chi-squared = 200.64, I-squared = 97%.

Bottom Summary:

The total 95% C I diamond for all 13 studies is centered at -1.03 with a range of -1.23 to -0.83, indicating a statistically significant improvement favoring post-treatment scores. The X-axis at the bottom ranges from -10 to 10, with 0 as the line of no effect. Labels indicate ‘Favours Post-treatment Sc’ to the left of zero and ‘Favours Pre-treatment Sc’ to the right.

Navigate back to Image 2:.

### Image 3: Long description

The table contains columns for Study or Subgroup, Stem Cells Group (Mean, S D, Total), Placebo Group (Mean, S D, Total), Weight, Mean Difference (I V, Fixed, 95% C I), and Year.

Data rows:

* Lv, 2013- C A R S: Stem Cells Mean 35.14, S D 7.77, Total 14; Placebo Mean 40.31, S D 3.82, Total 14; Weight 47.2%; Mean Difference -5.17 [-9.71, -0.63].

* Chez, 2018- V A B C: Stem Cells Mean 67.57, S D 16.52, Total 14; Placebo Mean 75, S D 11.6, Total 15; Weight 8.9%; Mean Difference -7.43 [-17.89, 3.03].

* Sharifzadeh, 2020- C A R S: Stem Cells Mean 30.75, S D 5, Total 14; Placebo Mean 35.06, S D 8.45, Total 18; Weight 43.9%; Mean Difference -4.31 [-9.01, 0.39].

Totals:

* Total (95% C I): Stem Cells Total 42; Placebo Total 47; Weight 100.0%; Mean Difference -4.99 [-8.11, -1.88].

* Heterogeneity: Chi super 2 = 0.30, d f = 2 (P = 0.86); I super 2 = 0%.

* Test for overall effect: Z = 3.14 (P = 0.002).

The forest plot on the right shows three green squares representing individual study effects and a black diamond representing the pooled effect. The x-axis ranges from -10 to 10, with 0 as the line of no effect. Values to the left of 0 are labeled Favours Stem Cell Group and values to the right are labeled Favours Placebo Group. The diamond is located entirely to the left of the zero line, indicating a statistically significant result favoring the Stem Cell Group.

Navigate back to Image 3:.

